# Impact of gender, lunge direction, and fatigue on the lower limb biomechanics in badminton lunges

**DOI:** 10.1371/journal.pone.0327428

**Published:** 2025-07-02

**Authors:** Zhonghao Xie, Jing Pan, Huiting Liang, Zhuoheng Wu, Jiayu Chen, Dongping Tan, Meng Wu, Zhiguan Huang

**Affiliations:** 1 Graduate School, Guangzhou Sport University, Guangzhou, China; 2 School of Educational Sciences, Guangdong Polytechnic, Guangzhou, China; 3 School of Competitive Sports, Guangdong Vocational Institute of Sport, Guangzhou, China; 4 Guangzhou Institute of Sports Science, Guangzhou, China; 5 School of Sports and Health, Guangzhou Sport University, Guangzhou, China; University of Castilla-La Mancha, SPAIN

## Abstract

**Background:**

The lunge is a crucial movement in badminton, influenced to different extents by factors such as lunge direction, fatigue, and gender. This study investigates the effects of these factors on lower limb biomechanics to inform strategies for injury prevention and performance optimization.

**Methods:**

Twenty-four amateur badminton players (12 males, 12 females) performed forehand and backhand forward lunges under both fatigued and non-fatigued conditions. Lower limb kinematics and kinetics were recorded using an 8-camera Vicon system and two AMTI force plates. A three-way mixed-design ANOVA was conducted to examine the effects of gender, lunge direction, and fatigue on biomechanical variables.

**Results:**

Forehand lunges demonstrated significantly higher peak joint moments at the hip (extension and external rotation), knee (internal rotation), and ankle (adduction) than backhand lunges. Under fatigued conditions, participants exhibited reduced knee flexion angles and increased leg stiffness. Regarding gender differences, females showed significantly higher knee internal rotation moments and vertical ground reaction force, while males exhibited greater ankle inversion angles. The interaction between gender and lunge direction revealed that males had a significantly smaller hip range of motion in the transverse plane than females, whereas males had greater knee coronal plane range of motion in backhand lunges.

**Conclusion:**

Lower limb movement strategies differ between forehand and backhand forward lunges, with backhand lunges potentially posing a greater risk of ankle injury. Fatigue reduces knee flexion and increases leg stiffness, which may elevate the load on the lower limbs. Gender differences also influence knee moments and ground reaction forces, with females experiencing higher loads. The interaction between gender and lunge direction revealed distinct movement patterns. Females may benefit from targeted hip and knee strengthening exercises to improve lunge performance and reduce injury risk.

## Introduction

Badminton is one of the most popular sports globally, welcoming participants of all ages, genders, and skill levels [1,2]. The sport demands quick directional changes, jumps, lunges at the net, and rapid arm movements from various postural positions [3]. Mastering effective footwork is essential for players to position themselves optimally for shots and swiftly return to the base position in preparation for opponents’ returns [4]. Among all footwork types, lunges are among the most common, constituting over 15% of all movements [3,4]. During lunges, the load of movements and impacts mainly falls on the dominant limb, causing players to exert greater eccentric efforts during the braking phase [5,6] and experience impact loads up to 2.5 times their body weight [2], necessitating high muscle activity and maintaining substantial core and knee dynamic stability to manage rapid changes in body position [2,7]. However, such demanding footwork increases the risk of injuries to the lower limbs [7,8]. Previous studies indicate that mastering the lunge helps to enhance performance and mitigate injury risks in badminton [7,9]. Consequently, conducting an in-depth study on badminton lunge may help to provide scientific information for the prevention of lower limb injuries.

Lunges can be categorized into four primary directions [3]. Among these, the forehand and backhand forward lunges are considered the most critical, as they produce the highest plantar loading compared to other lunge directions [3,10]. Forward lunges in different directions exhibit distinct dynamic characteristics [3,11]. Specifically, Hong et al. [3] reported that backhand forward lunges generate higher ground reaction forces and plantar pressures in right-handed badminton players compared to forehand lunges, suggesting a greater injury risk during backhand lunges in male players. Conversely, Nielsen et al. [11] found that backhand lunges are associated with significantly lower hip, knee, and ankle frontal plane moments than forehand lunges in male players, potentially indicating a reduced risk of overuse injuries and discomfort. However, Xie et al. [12] found no significant difference in frontal plane moments between forehand and backhand lunges in female amateur players. These conflicting results may be due to differences in gender and skill level, indicating that considering these factors in further lunge studies may help to understand the kinematic and kinetic differences between different lunge directions.

Gender differences are an important factor influencing lunge performance. These differences become apparent during puberty, alongside an increase in endogenous sex hormones, particularly testosterone in males [13]. Males generally possess larger, stronger, faster, and more powerful skeletal muscles than females [14]. Studies on gender have indicated that female players face higher injury risks compared to their male counterparts [15,16]. Specifically, Lam et al. [17] examined badminton players of varying genders and skill levels and found that unskilled female players—defined as those without formal competition experience and with only two to three years of training—experienced higher impact forces during backhand forward lunges, making them more susceptible to lower-limb injuries. These findings highlight the need for further biomechanical studies focusing on unskilled female players to better understand injury mechanisms and inform targeted training interventions. Additionally, since forward lunges in different directions exhibit distinct dynamic characteristics [3,11], whether the same gender differences are observed in forehand forward lunges still requires further exploration.

Beyond lunge direction and gender difference, fatigue significantly impacts lunge performance. Fatigue is defined as a reduction in force production capacity, irrespective of the performed movements [18–20], arising from physiological, mechanical, and psychological changes [21]. It adversely affects both performance and injury risk by diminishing joint position sense [22], increasing muscle stiffness [23], altering lower limb biomechanics, and reducing dynamic posture control [20]. For instance, performing repeated lunges until fatigue significantly alters the activity of the vastus lateralis and biceps femoris [24], increases joint stiffness and elevates injury risk [25]. Additionally, fatigue of the ankle dorsiflexors adversely affects forehand lunge performance and increases the impact force during backhand lunges [26]. Factors such as training density and duration [27], anatomical movement positions [28], and duration of concentric/eccentric movements all result in distinct muscle activation and neuromuscular fatigue responses. Therefore, the chosen fatigue protocol is crucial for accurately interpreting results. Badminton is characterized by actions of short duration and high intensity coupled with short rest periods [2,29]. Considering these characteristics, a sprint interval running and maximal vertical jump fatigue protocol [30,31] may help simulate the fatigue experienced during a match in the laboratory.

To sum up, forward lunges in different directions exhibit distinct biomechanical characteristics [3,11], players of different genders exhibit distinct biomechanical characteristics during lunges [17] and fatigue negatively impacts biomechanical characteristics during lunges [26]. This shows that there are mutual connections and influences among lunge direction, gender differences, and fatigue. However, few lunge studies have simultaneously examined lunge direction, fatigue, and gender differences. The interaction of these factors is still not clear. A comprehensive analysis of these three factors on the lower limb biomechanics during lunges may enhance our understanding of this movement and help to provide a scientific training strategy.

To address these gaps, this study recruited male and female amateur badminton players to perform forehand and backhand forward lunges on a simulated badminton court within a laboratory setting. We collected kinematic and dynamic data of the lower limbs under both fatigued and non-fatigued conditions. The primary objective was to investigate how lunge direction, fatigue, and gender affect the kinematics and dynamics of the lower limbs during badminton lunges. Additionally, we aim to evaluate the potential interactions among these factors and their combined impact on lower limb performance. We hypothesize that each factor will distinctly influence lower limb biomechanics and that their interactions may further affect performance and injury risk in badminton.

## Materials and methods

### Participants

To ensure sufficient statistical power, the sample size was calculated using G*Power 3.1 software, with an effect size (f) of 0.25, an alpha of 0.05, and a power of 0.8, indicating a minimum requirement of 24 participants. Accordingly, 24 amateur badminton players (12 males and 12 females) from Guangzhou Sport University were recruited. Participants were classified as amateur players based on their non-professional status and engagement in recreational badminton activities [12].

The male participants had a mean height of 1.75 ± 0.04 m, a weight of 65.33 ± 6.4 kg, and a BMI of 21.43 ± 1.84 kg/m², whereas the female participants averaged 1.65 ± 0.03 m in height, 51.5 ± 4.08 kg in weight, and a BMI of 18.9 ± 1.43 kg/m².

Inclusion criteria were as follows: (1) age between 18 and 24 years; (2) right-handedness; (3) regular badminton practice (at least 6 hours per week); (4) a minimum of 2 years of playing experience; (5) no formal competition experience; and (6) no history of lower limb injury in the past year.

Ethical approval was obtained from the Ethics Committee of Guangzhou Sport University (approval number 2024LCLL-106). All participants signed a written informed consent form before the test and refrained from strenuous exercise for 24 hours prior to the experiment.

### Experimental setup

This experiment took place at the Guangdong Sports Equipment Engineering Technology Research Center from October 26, 2024, to November 27, 2024. Each participant’s data collection session lasted about 90 minutes, including warm-up, equipment setup, and data recording.

**Fig 1:**
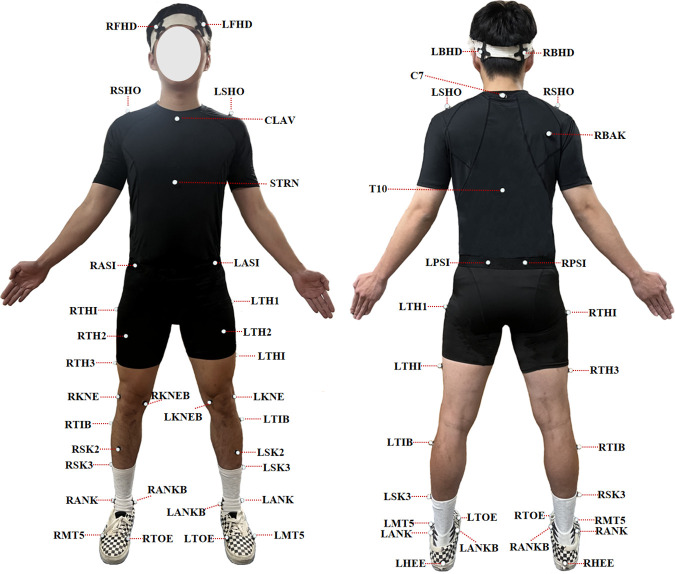
Subject markers protocol. The marker placement locations included the following anatomical landmarks: the brow bone, occipital bone, acromion, C7 vertebra, center of the right scapula, T10 vertebra, center of the clavicle, lowest point of the sternum, anterior and posterior superior iliac spines, medial and lateral femoral condyles, lateral thigh, lateral calf, medial and lateral malleoli, heel, and the first and fifth metatarsal heads.

Kinematic measurements: An 8-camera Vicon motion capture system (Oxford Metrics Ltd., Oxford, UK) operating at 200 Hz was used to collect raw kinematic data during the badminton lunge movements. Forty-one reflective markers were placed on specific anatomical landmarks (Fig 1). Kinetic measurements: The raw kinetic data were collected using two AMTI force platforms (Watertown, MA, United States) operating at 1000 Hz. These platforms were embedded flush with the floor of the laboratory-simulated badminton court to capture kinetic data during the lunge phases.

### Experimental procedure

Test preparation phase: Participants were first briefed on the test procedures and safety precautions. To standardize the lunge distance [9], start and end points were marked on the floor at 1.5 times each participant’s leg length (measured from the ASIS to the ground). They then completed a 5–10-minute warm-up session, which included dynamic stretching and practice lunges, before donning Suunto heart rate belts (Suunto Oy, Vantaa, Finland) for continuous heart rate monitoring throughout the experiment.

**Fig 2:**
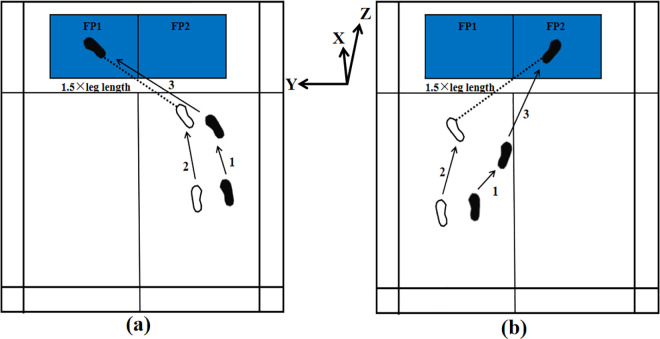
Schematic diagram of forehand and backhand forward lunges. (a) indicates the backhand forward lunge, and (b) represents the forehand forward lunge. The dashed line represents 1.5 times the leg length. The X, Y, and Z coordinates are defined according to the force plate. White footprints indicate the position of the left foot, and black footprints indicate the position of the right foot, with numbers indicating the step sequence. FP1 and FP2 represent two different force plates.

Formal testing phase: participants performed a three-step lunge technique, incorporating both forehand and backhand lunges (Fig 2). The formal testing comprised two conditions: pre-fatigue and post-fatigue. Pre-fatigue testing: participants started from a designated position and executed the lunge as powerfully as possible, ensuring their dominant leg landed on the center of the force platform, then returned quickly to the starting point. Each participant completed five valid trials of both forehand and backhand lunges in a randomized order, with a 30-second rest interval between trials. A trial was deemed valid if the motion was executed correctly and the foot fully contacted the force platform without targeting errors.

**Fig 3:**
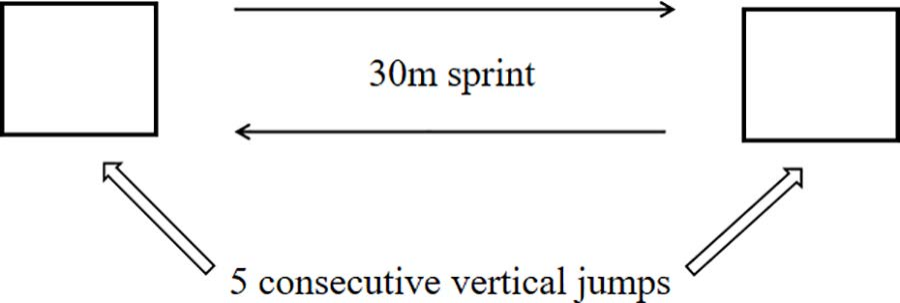
Fatigue Intervention. This fatigue exercise consisted of unlimited repetitions of 5 consecutive vertical jumps (each averaging over 70% of their maximum vertical jump heights) followed by a 30-m sprint (accelerate and decelerate as quickly as possible). This process is repeated until the fatigue criteria are met.

Fatigue intervention: a sprint interval running and maximal vertical jump protocol [31] (Fig 3) was used. Fatigue was considered achieved when (1) consecutive vertical jumps fell below 70% of the maximum jump height, (2) the Rate of Perceived Exertion (RPE) scale reached 17 or higher, or (3) heart rate reached 80% of the participant’s maximum. Once these criteria were met, the intervention ceased.

Post-fatigue testing: immediately afterward, participants performed five valid forehand and backhand lunge trials under the post-fatigue condition, following the same randomized order and validity criteria used in pre-fatigue testing. No rest intervals were given to ensure the fatigue state was maintained.

### Data reduction

**Fig 4:**
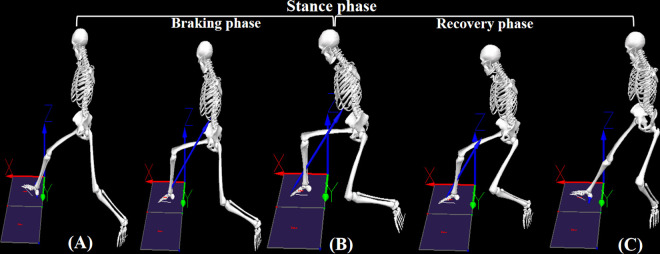
Division of lunge phases. The forehand forward lunge is used as an example to illustrate the division of lunge phases: (A) Initial contact: the heel contacts the force plate, with vertical ground reaction force > 15 N; (B) Moment of minimum flexion angle in the dominant knee joint; (C) Lift-off: the dominant leg leaves the force plate, with vertical ground reaction force < 15 N. The braking phase is defined as the period from (A) to (B). The recovery phase is defined as the period from (B) to (C). The stance phase is defined as the period from (A) to (C) [10].

Raw kinematic and kinetic data were imported into Visual3D software (C-Motion Inc., Rockville, MD, USA) for processing. Kinematic and kinetic data were filtered using a fourth-order Butterworth filter with cutoff frequencies of 15 and 25 Hz, respectively [32]. The lunge movement was divided into braking, recovery, and stance phases (Fig 4). This study focused on changes in biomechanical parameters of the racket-holding side lower limb across these phases.

The following kinematic variables were calculated: joint (hip, knee, ankle) angles and joint range of motion (ROM). Kinetic variables included ground reaction force (GRF), joint moment, mean loading rate, maximum loading rate, leg stiffness [33,34] (Equation 1), and joint stiffness [33,34] (Equation 2).


$$Kleg=\frac{PeakGRF}{\Delta L}$$ (1)

(where K_leg_ = leg stiffness, PeakGRF = the maximum vertical ground reaction force; ΔL = the maximum change in vertical leg length)


$$Kjoint=\frac{\Delta M}{\Delta \theta }$$ (2)

(where K_joint _= joint stiffness, ΔM = change in joint moment; Δθ = change in joint angle)

**Fig 5:**
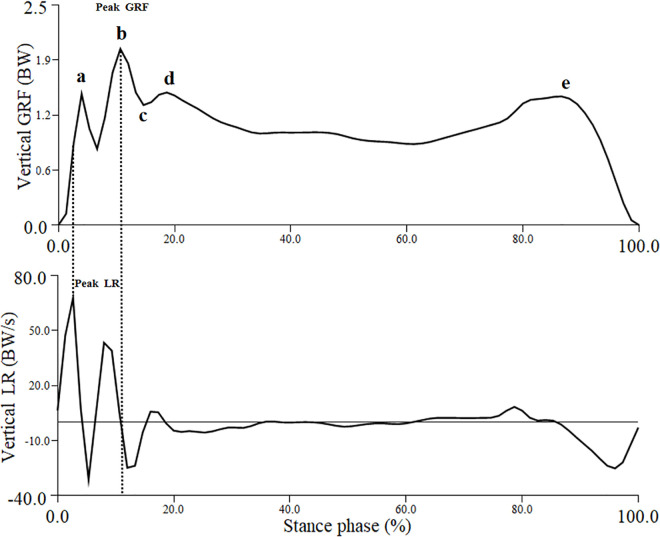
Example identification of phases from vertical loading rate and vertical ground reaction force. The five force phases (a, b, c, d, and e) are identified. BW = body weight; GRF = ground reaction force; LR = loading rate.

In the GRF profile of a badminton lunge, five stages have been identified [9,11]: initial impact peak (heel strike transient), secondary impact peak (impact loading), amortization, weight acceptance, and drive-off (Fig 5). This study focuses on analyzing the vertical GRF at each of these stages to better understand load distribution. The mean loading rate was calculated from the force slope during the period in which the force increased from 20% to 90% of the initial peak magnitude [35]. The maximum loading rate was defined as the maximum value reached by the stress/force slopes during every 1% of the stance period from initial contact to initial peak force [35]. All kinetic data were normalized to each participant’s body weight (BW). The mean of three valid trials for each variable was then used for analysis.

### Statistical analysis

All variables were expressed as mean ± standard deviation (Mean ± SD). The Shapiro–Wilk normality test and Levene’s test were performed to evaluate data normality and homogeneity of variance, respectively. A three-factor mixed design ANOVA (lunge direction × fatigue × gender, 2 × 2 × 2) was used to analyze the effects of these factors on lower limb biomechanical parameters. All statistical analyses were conducted using SPSS v.17.0 (IBM, Armonk, NY, USA), and effect sizes were reported as η². The significance level was set at p < 0.05. When significant interaction effects were observed, the Bonferroni correction was applied to control for multiple comparisons by adjusting the significance level for post-hoc pairwise tests.

## Results

### Joint angle

Gender, fatigue, and lunge direction did not significantly affect the peak flexion or adduction angles of the hip joint. However, fatigue had a highly significant effect on the hip’s peak internal rotation angle (P = 0.004, F = 8.66, η² = 0.09). Post hoc analysis showed that under non-fatigued conditions (18.69 ± 8.84°), the hip’s peak internal rotation angle was significantly greater than under fatigued conditions (13.85 ± 7.43°). Fatigue also significantly affected the peak flexion angle of the knee joint (P = 0.028, F = 5.02, η² = 0.05), where non-fatigued conditions (−81.71 ± 8.63°) showed a greater flexion angle than fatigued conditions (−77.51 ± 8.30°).

In contrast, lunge direction significantly affected the ankle’s peak dorsiflexion angle (P = 0.009, F = 7.25, η² = 0.08). Post hoc analysis indicated that forehand lunges (8.17 ± 4.43°) had a smaller dorsiflexion angle than backhand lunges (10.79 ± 4.83°). Furthermore, both gender (P = 0.011, F = 6.75, η² = 0.71) and direction (P < 0.01, F = 72.31, η² = 0.45) significantly influenced the ankle’s peak inversion angle. Male participants (18.17 ± 8.77°) exhibited a greater inversion angle than female participants (15.29 ± 5.38°), and backhand lunges (21.43 ± 6.53°) resulted in a greater inversion angle than forehand lunges (12.03 ± 4.72°) (Table 1).

**Table 1:** Effects of gender, direction, and fatigue on the mean (± SD) lower-limb peak joint angles during the stance phase.

Variables		Female				Male			
Peak joint angle (°)		Forehand		Backhand		Forehand		Backhand	
		pre	post	pre	post	pre	post	pre	post
**Hip**	**flexion**	92.87 ±13.19	90.73 ±14.55	96.6 ±13.13	93.4 ±15.55	98.5 ±14.47	90.9 6 ± 17.7	99.51 ±15.8	92.49 ±16.46
	**adduction**	11.67 ±3.3	11.6 5 ± 4.76	18.86 ±3.83	18.89 ±4.23	12.49 ±6.41	12.31 ±4.46	26.55 ±8.55	21.32 ±5.87
	**internal Rotation**	**13.64** **±7.42** ^**c**^	**12.23** **±8.45** ^**c**^	**18.66** **±8.77** ^**c**^	**16.06** **±7.62** ^**c**^	**21.29** **±9.45** ^**c**^	**13.42** **±7.27** ^**c**^	**21.16** **±8.41** ^**c**^	**13.68** **±6.71** ^**c**^
**Knee**	**flexion**	**−81.2** **±8.56** ^**c**^	**78.41** **±7.74** ^**c**^	**−81.2** **±8.37** ^**c**^	**79.9** **±10.4** ^**c**^	**−81.27** **±8.37** ^**c**^	**−77.2** **±7.06** ^**c**^	**−81.6** **±10.4** ^**c**^	**−74.89** **±7.95** ^**c**^
	**varus**	13.54 ±6.71	11.59 ±5.76	13.68 ±5.55	11.6 ±6.08	15.91 ±9.8	12.14 ±10.8	11.07 ±10.6	8.95 ±8
	**external rotation**	−22.88 ±7.16	−24.52 ±7.9	−23.31 ±8.85	−22.84 ±8.14	−21.26 ±13.02	−20.7 ±11.49	−17.1 ±14.43	19.22 ±12.66
**Ankle**	**dorsi-flenxion**	**8.24** **±6.06** ^**b**^	**8.86** **±5.37** ^**b**^	**10.94** **±5.74** ^**b**^	**11.18** **±5.34** ^**b**^	**7.4** **±2.38** ^**b**^	**8.15** **±3.41** ^**b**^	**10.65** **±5.02** ^**b**^	**10.37** **±3.52** ^**b**^
	**inversion**	**11.67** **±3.3** ^**ab**^	**11.65** **±4.76** ^**ab**^	**18.96** **±3.83** ^**ab**^	**18.89** **±4.23** ^**ab**^	**12.49** **±6.4** ^**ab**^	**12.49** **±6.41** ^**ab**^	**26.55** **±8.6** ^**ab**^	**21.32** **±5.87** ^**ab**^
	**external Rotation**	−13.92 ±8.57	−12.91 ±8.85	−14.32 ±8.6	−13.98 ±9.3	−15.69 ±6.56	−14.78 ±6.3	−18.58 ±6.6	−15.91 ±6.54

^a^Denotes a statistically significant difference between genders (P < 0.05).

^b^Denotes a statistically significant difference between movement directions (P < 0.05).

^c^Denotes a statistically significant difference between pre-fatigue and post-fatigue conditions (P < 0.05).

### Joint range of motion

**Fig 6:**
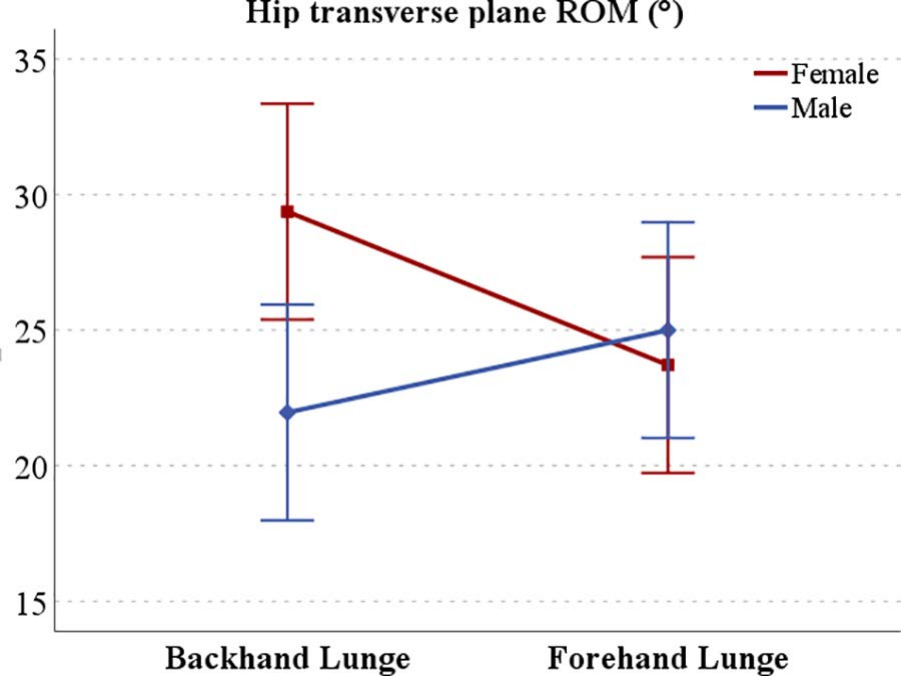
Interaction effect of gender and lunge direction on hip transverse plane range of motion (ROM). Error bars represent 95% confidence intervals.

Hip range of motion: fatigue significantly affected the hip’s transverse plane ROM (p = 0.044, F = 3.91, η² = 0.04), with pre-fatigue values (26.99 ± 11.43°) exceeding post-fatigue (23.03 ± 8.12°). Additionally, a significant interaction between gender and direction was found (p = 0.033, F = 3.72, η² = 0.05), and gender had a significant effect in backhand lunges (p = 0.010, F = 6.85, η² = 0.07) (Fig 6). Post-hoc analysis showed that, in backhand lunges, males (21.96 ± 11.06°) had a smaller transverse plane ROM than females (29.38 ± 8.77°). Lunge direction also significantly affected females (p = 0.049, F = 3.99, η² = 0.04), whose hip transverse ROM was lower in forehand lunges (23.72 ± 8.64°) compared to backhand lunges (29.38 ± 8.77°).

Knee range of motion: fatigue significantly impacted the knee coronal plane ROM (p = 0.041, F = 4.32, η² = 0.05), with pre-fatigue values (21.09 ± 13.02°) exceeding post-fatigue (16.84 ± 6.28°). A significant gender–direction interaction was also observed (p = 0.040, F = 4.36, η² = 0.03). Specifically, in males, lunge direction significantly influenced the knee’s coronal plane (p = 0.050, F = 3.84, η² = 0.04), where forehand lunges (21.77 ± 18.29°) exhibited a smaller ROM than backhand lunges (16.09 ± 6.24°).

Ankle range of motion: lunge direction significantly affected the ankle’s sagittal plane (p = 0.033, F = 4.68, η² = 0.05) and transverse plane (p = 0.005, F = 8.44, η² = 0.09). Post-hoc analysis revealed that the sagittal plane ROM in forehand lunges (33.32 ± 8.96°) was smaller than in backhand lunges (37.75 ± 12.51°). Similarly, the transverse plane ROM for forehand lunges (13.54 ± 4.39°) was lower than for backhand lunges (16.53 ± 5.59°). Gender significantly influenced the ankle’s coronal plane (p = 0.010, F = 6.96, η² = 0.07), with males (17.78 ± 8.11°) displaying a larger ROM than females (14.31 ± 4.20°) (Table 2).

**Table 2:** Effects of gender, direction, and fatigue on the mean (± SD) lower-limb range of motion during the stance phase.

Variables		Female				Male			
Joint range of motion (°)		Forehand		Backhand		Forehand		Backhand	
		pre	post	pre	post	pre	post	pre	post
**Hip**	**Sagittal**	50.79 ±8.24	48.56 ±11.89	53.06 ±9.34	51.2 ±14.34	50.93 ±21.6	50.46 ±14.6	56.4 ±13.91	50.15 ±13.8
	**Coronnal**	20.94 ±7.5	19.83 ±6.88	20.52 ±7.46	19.15 ±7.3	28.02 ±15.54	20.39 ±6.61	21.5 ±8.47	20.46 ±5.43
	**Transverse**	**24.58** **±10.4** ^**cd**^	**22.85** **±6.88** ^**cd**^	**31.1** **±8.6** ^**cd**^	**27.7** **±8.95** ^**cd**^	**27.75** **±12.6** ^**cd**^	**22.3** **±7.8** ^**cd**^	**24.6** **±13.7** ^**cd**^	**19.33** **±7.3** ^**cd**^
**Knee**	**Sagittal**	69.92 ±8.95	67.63 ±7.01	70.79 ±8.42	69.77 ±10.4	64.24 ±22.7	69.55 ±9.38	75.67 ±12.1	67.59 ±10.2
	**Coronnal**	**17.79** **±5.81** ^**cd**^	**17.36** **±5** ^**cd**^	**20.9** **±4.2** ^**cd**^	**19.96** **±5.6** ^**cd**^	**27.8** **±23.6** ^**cd**^	**15.73** **±8** ^**cd**^	**17.86** **±6.7** ^**cd**^	**14.34** **±5.4** ^**cd**^
	**Transverse**	20.06 ±6.73	20.86 ±5.66	22.47 ±5.37	21.35 ±5.48	23.18 ±6.17	20.02 ±3.81	23.82 ±6.7	22.2 ±6.03
**Ankle**	**Sagittal**	**33.95** **±10.9** ^**b**^	**33.49** **±8.96** ^**b**^	**38.7** **±10.9** ^**b**^	**36.47** **±11.5** ^**b**^	**32.23** **±9.57** ^**b**^	**33.64** **±7.2** ^**b**^	**39.34** **±10.6** ^**b**^	**36.5** **±10.14** ^**b**^
	**Coronnal**	**13.93** **±4.1** ^**a**^	**13.33** **±4.77** ^**a**^	**14.91** **±4.3** ^**a**^	**15.1** **±3.94** ^**a**^	**17.16** **±9.44** ^**a**^	**15.06** **±5.2** ^**a**^	**21.54** **±9.28** ^**a**^	**17.34** **±7.36** ^**a**^
	**Transverse**	**14.21** **±5.9** ^**b**^	**14.26** **±5.7** ^**b**^	**15.66** **±5.6** ^**b**^	**16.8** **±6.16** ^**b**^	**13.62** **±2.25** ^**b**^	**12.06** **±2.6** ^**b**^	**18.68** **±6.28** ^**b**^	**14.98** **±4.08** ^**b**^

^a^Denotes a statistically significant difference between genders (P < 0.05).

^b^Denotes a statistically significant difference between movement directions (P < 0.05).

^c^Denotes a statistically significant difference between pre-fatigue and post-fatigue conditions (P < 0.05).

^d^Denotes a statistically significant interaction effect between gender and movement direction (P < 0.05).

### Joint moment

Hip Joint Moment: Lunge direction had a significant effect on the peak hip extension, adduction, and external rotation moments (extension: p = 0.003, F = 9.152, η² = 0.094; adduction: p = 0.025, F = 5.225, η² = 0.056; external rotation: p < 0.001, F = 13.76, η² = 0.135). Post hoc analysis revealed that the peak hip extension moment during forehand lunges (−4.07 ± 0.86 Nm/kg) was significantly greater than during backhand lunges (−3.56 ± 0.65 Nm/kg). Additionally, the peak hip external rotation moment during forehand lunges (−1.32 ± 0.42 Nm/kg) was significantly greater than during backhand lunges (−1.01 ± 0.44 Nm/kg). Conversely, the peak hip adduction moment during forehand lunges (2.05 ± 0.82 Nm/kg) was significantly smaller than during backhand lunges (2.42 ± 0.74 Nm/kg). Furthermore, gender had a significant effect on the peak hip adduction (p = 0.004, F = 8.699, η² = 0.09) and external rotation moments (p = 0.014, F = 6.248, η² = 0.066). Specifically, males exhibited a higher peak hip adduction moment (2.47 ± 0.82 Nm/kg) compared to females (1.99 ± 0.76 Nm/kg), while females demonstrated a greater peak hip external rotation moment (−1.27 ± 0.45 Nm/kg) than males (−1.05 ± 0.44 Nm/kg).

Knee Joint Moment: Lunge direction significantly affected the peak knee extension, adduction, and internal rotation moments (extension: p = 0.006, F = 7.952, η² = 0.083; adduction: p < 0.01, F = 15.75, η² = 0.15; internal rotation: p < 0.01, F = 35.5, η² = 0.28). Post hoc analysis indicated that the peak knee extension moment during backhand lunges (2.46 ± 0.51 Nm/kg) was significantly greater than during forehand lunges (2.10 ± 0.71 Nm/kg). Similarly, the peak knee adduction moment during backhand lunges (1.21 ± 0.58 Nm/kg) was significantly higher than during forehand lunges (0.58 ± 0.31 Nm/kg). In contrast, the peak knee internal rotation moment during forehand lunges (0.67 ± 0.28 Nm/kg) was significantly greater than during backhand lunges (0.33 ± 0.28 Nm/kg). Additionally, gender had a significant effect on the knee internal rotation moment (p = 0.044, F = 4.176, η² = 0.045), with females exhibiting a higher peak knee internal rotation moment (0.56 ± 0.35 Nm/kg) compared to males (0.44 ± 0.30 Nm/kg).

Ankle Joint Moment: Lunge direction had a significant effect on the peak ankle inversion and eversion moments (inversion: p < 0.01, F = 16.26, η² = 0.156; eversion: p < 0.01, F = 21.998, η² = 0.200). Post hoc tests showed that the peak ankle inversion moment during backhand lunges (0.42 ± 0.10 Nm/kg) was significantly greater than during forehand lunges (0.26 ± 0.47 Nm/kg). Additionally, the peak ankle eversion moment during forehand lunges (0.28 ± 0.25 Nm/kg) was significantly higher than during backhand lunges (0.10 ± 0.09 Nm/kg) (see Table 3).

**Table 3:** Effects of gender, direction, and fatigue on mean (± SD) lower-limb peak moments during the stance phase.

Variables		Female				Male			
Peak joint moment (Nm/kg)		Forehand		Backhand		Forehand		Backhand	
		pre	post	pre	post	pre	post	pre	post
**Hip**	**Extension**	**−3.97** **±1.28** ^**b**^	**−3.89** **±1.02** ^**b**^	**−3.47** **±0.74** ^**b**^	**−3.45** **±0.69** ^**b**^	**−4.41** **±0.62** ^**b**^	**−4.01** **±0.82** ^**b**^	**−3.81** **±0.47** ^**b**^	**−3.51** **±0.68** ^**b**^
	**Adduction**	**1.88** **±0.69** ^**ab**^	**1.62** **±0.65** ^**ab**^	**2.28** **±0.81** ^**ab**^	**2,21** **±0.82** ^**ab**^	**2.56** **±1.09** ^**ab**^	**2.14** **±0.77** ^**ab**^	**2.63** **±0.74** ^**ab**^	**2.54** **±0.6** ^**ab**^
	**External rotation**	**−1.51** **±0.54** ^**ab**^	**−1.44** **±0.4** ^**ab**^	**−1.05** **±0.42** ^**ab**^	**−1.09** **±0.24** ^**ab**^	**−1.15** **±0.3** ^**ab**^	**−1.19** **±0.33** ^**ab**^	**−0.89** **±0.56** ^**ab**^	**−0.97** **±0.5** ^**ab**^
**Knee**	**Extension**	**2.2** **±0.61** ^**b**^	**2.16** **±0.58** ^**b**^	**2.68** **±0.41** ^**b**^	**2.54** **±0.41** ^**b**^	**2.05** **±0.88** ^**b**^	**2.01** **±0.82** ^**b**^	**2.44** **±0.55** ^**b**^	**2.19** **±0.59** ^**b**^
	**Varus**	**0.5** **±0.25** ^**b**^	**0.41** **±0.2** ^**b**^	**1.15** **±0.67** ^**b**^	**−0.41** **±0.29** ^**b**^	**0.78** **±0.33** ^**b**^	**0.64** **±0.34** ^**b**^	**1.35** **±0.57** ^**b**^	**1.27** **±0.58** ^**b**^
	**Internal rotation**	**0.7** **±0.32** ^**ab**^	**0.58** **±0.25** ^**ab**^	**1.05** **±0.32** ^**ab**^	**1.09** **±0.45** ^**ab**^	**0.84** **±0.33** ^**ab**^	**0.79** **±0.26** ^**ab**^	**1.19** **±0.4** ^**ab**^	**1.27** **±0.36** ^**ab**^
**Ankle**	**Plantar-flexion**	−1.53 ±0.66	−1.5 1 ± 0.63	−1.42 ±0.48	−1.46 ±0.6	−1.44 ±0.59	−1.46 ±0.56	−1.41 ±0.34	−1.36 ±0.36
	**Inversion**	**0.11** **±0.07** ^**b**^	**0.1** **±0.07** ^**b**^	**0.47** **±0.51** ^**b**^	**0.41** **±0.5** ^**b**^	**0.19** **±0.12** ^**b**^	**0.16** **±0.11** ^**b**^	**0.43** **±0.46** ^**b**^	**0.4** **±0.47** ^**b**^
	**Adduction**	**0.32** **±0.29** ^**b**^	**0.33** **±0.33** ^**b**^	**0.08** **±0.07** ^**b**^	**0.07** **±0.07** ^**b**^	**0.25** **±0.19** ^**b**^	**0.24** **±0.17** ^**b**^	**0.14** **±0.12** ^**b**^	**0.12** **±0.09** ^**b**^

^a^Denotes a statistically significant difference between genders (P < 0.05).

^b^Denotes a statistically significant difference between movement directions (P < 0.05).

### Ground reaction force and loading rates

Results indicated a significant gender effect on vertical ground reaction force (GRF) during the secondary impact peak phase, with females showing a significantly greater GRF (1.75 ± 0.21 BW) than males (1.65 ± 0.17 BW) (p = 0.01, F = 6.36, η² = 0.07). No other stages demonstrated statistically significant differences in GRF between genders. Furthermore, lunge direction, gender, and fatigue status did not have significant effects on the average or maximum loading rates (Table 4).

**Table 4:** Effects of gender, direction, and fatigue on the mean (± SD) values of GRF, loading rate (LR), joint stiffness, and leg stiffness.

Variables	Female				Male			
GRF, LR, and stiffness	Forehand		Backhand		Forehand		Backhand	
	pre	post	pre	post	pre	post	pre	post
**Initial impact peak** **(BW)**	1.63 ±0.25	1.61 ±0.29	1.67 ±0.26	1.67 ±0.25	1.62 ±0.31	1.51 ±0.27	1.71 ±0.32	1.65 ±0.42
**Secondary** **impact peak (BW)**	**1.75** **±0.2** ^**a**^	**1.72** **±0.16** ^**a**^	**1.8** **±0.24** ^**a**^	**1.74** **±0.23** ^**a**^	**1.71** **±0.19** ^**a**^	**1.61** **±0.16** ^**a**^	**1.68** **±0.15** ^**a**^	**1.63** **±0.17** ^**a**^
**Amortization (BW)**	1.38 ±0.17	1.36 ±0.18	1.4 ±0.2	1.37 ±0.22	1.34 ±0.19	1.28 ±0.2	1.3 ±0.29	1.3 ±0.24
**Weight** **Acceptance (BW)**	1.46 ±0.13	1.43 ±0.15	1.49 ±0.22	1.48 ±0.24	1.5 ±0.19	1.39 ±0.22	1.46 ±0.25	1.41 ±0.25
**Drive-off (BW)**	1.52 ±0.15	1.47 ±0.11	1.5 ±0.18	1.5 ±0.18	1.45 ±0.14	1.44 ±0.11	1.51 ±0.19	1.53 ±0.17
**Mean** **loading rate (BW/s)**	21.61 ±10.7	24.21 ±13.33	26.1 ±10.5	27.45 ±9.76	26.8 ±13.26	20.1 ±10.01	28.74 ±15.1	27.41 ±13.4
**Maximum** **loading rate (BW/s)**	63.35 ±13.9	64.19 ±15.6	67.1 ±14.6	65.9 ±13.3	72.8 ±12.9	65.9 ±13.2	74.1 ±14.4	71.29 ±18.8
**Hip** **Stiffness (Nm/kg/°)**	**0.09** **±0.03** ^**b**^	**0.1±** **0.04** ^**b**^	**0.08** **±0.02** ^**b**^	**0.08** **±0.02** ^**b**^	**0.08** **±0.02** ^**b**^	**0.1** **±0.03** ^**b**^	**0.08** **±0.02** ^**b**^	**0.09** **±0.03** ^**b**^
**Knee** **stiffness (Nm/kg/°)**	0.05 ±0.01	0.05 ±0.01	0.05 ±0.01	0.05 ±.0.1	0.05 ±0.02	0.04 ±0.01	0.05 ±0.01	0.05 ±.0.1
**Ankle** **stiffness(Nm/kg/°)**	0.07 ±0.03	0.06 ±0.02	0.06 ±0.02	0.06 ±0.02	0.06 ±0.01	0.06 ±0.02	0.06 ±0.01	0.06 ±0.02
**Braking phase** **leg stiffness(BW/m)**	**12.56** **±3.2** ^**c**^	**14.2** **±5.56** ^**c**^	**13.52** **±2.7** ^**c**^	**13.97** **±3.66** ^**c**^	**14.22** **±4.9** ^**c**^	**14.1** **±3.5** ^**c**^	**14.1** **±5.1** ^**c**^	**17.88** **±9.04** ^**c**^
**Recovery phase** **leg stiffness (BW/m)**	9.22 ±2.1	9.64 ±2.2	9.63 ±3.08	10.45 ±3.89	8.6 ±2.04	10.47 ±4.5	8.68 ±2.1	11.39 ±3.94

^a^Denotes a statistically significant difference between genders (P < 0.05).

^b^Denotes a statistically significant difference between movement directions (P < 0.05).

^c^Denotes a statistically significant difference between pre-fatigue and post-fatigue conditions (P < 0.05).

### Stiffness

Joint Stiffness: Lunge direction significantly affected hip stiffness (p = 0.04, F = 4.5, η² = 0.05), with forehand lunges showing greater hip stiffness (0.09 ± 0.004 Nm/kg/°) compared to backhand lunges (0.08 ± 0.004 Nm/kg/°). Additionally, during the recovery phase, a significant difference in leg stiffness was observed based on fatigue state (p = 0.03, F = 5.22, η² = 0.06), where non-fatigued leg stiffness (9.03 ± 2.33 BW/m) was significantly lower than fatigued leg stiffness (10.49 ± 3.65 BW/m) (Table 4).

## Discussion

This study found that the direction of the lunge, gender, and fatigue status significantly affected the biomechanical characteristics of lower limbs during badminton lunges. Specifically, the direction of the lunge has a significant impact on the peak moment, hip stiffness, and ROM of the hip, knee, and ankle joints. Fatigue changes the ROM and peak angle of the hip and knee joints, and increases the leg stiffness in the recovery phase. Gender affects secondary impact peaks and joint moments of the knee and hip. In addition, there is a significant interaction between gender and the direction of lunges. Comprehensively understanding the biomechanical differences of lower limbs in badminton lunges may help to provide a scientific basis for injury prevention and competitive training strategies.

### Effects of lunge direction

Greater foot loading has been observed in forehand and backhand forward lunges, highlighting two critical directions for research [3,10,29]. In this study, the forehand forward lunge exhibited significantly larger peak joint moments at the hip (extension, external rotation), knee (internal rotation), and ankle (adduction). Moreover, the backhand forward lunge had larger peak joint moments at the hip (adduction), knee (extension, adduction), and ankle (inversion). This is different from the previous studies on professional athletes in different directional forward lunges [11], which participants experienced hip and knee transverse moments during backhand forward lunges, while hip, knee, and ankle frontal moments were higher during forehand lunges, which might imply that backhand forward lunge would be of lower risk of overuse injuries and discomfort. These differences might be a manifestation of the amateur players’ lack of proficiency in the lunge and poor control of the movement, showing greater differences in the lower limb movement strategies of the amateur players when performing the forehand and backhand forward lunges. Therefore, the statement that “backhand forward lunge would be of lower risk of overuse injuries and discomfort” might not be applicable to amateur badminton players. It is suggested that amateur players need to improve their skills and increase strength training to cope with such highly demanding movements.

Ankle injuries are the most common among all lower-limb injuries in badminton, accounting for over half of reported cases [36], with more than 80% classified as lateral ankle sprains (LAS) [37,38]. LAS causes structural damage to the lateral collateral ligaments (anterior tibiofibular ligament and calcaneofibular ligament) of the ankle joint [37,38]. These structural injuries may cause joint pain, swelling, and related dysfunction [39]. Biomechanically, increased ankle inversion angles and moments are recognized as key risk factors contributing to LAS [37,38,40]. In the present study, both peak ankle inversion angles and moments were significantly greater during backhand lunges compared with forehand lunges, suggesting a heightened risk of LAS during backhand movements in amateur players.

Furthermore, the ankle’s range of motion (ROM) in the sagittal plane was found to be greater during backhand lunges. This may also contribute to LAS risk. Prior studies have demonstrated that increased ankle sagittal plane ROM and greater initial contact angles during single-leg landings enhance joint energy dissipation and reduce impact loads on the lower limbs, thereby mitigating the risk of injuries such as anterior cruciate ligament (ACL) tears [39]. However, in the absence of strengthened tissues and muscles surrounding the ankle to accommodate the increased ankle sagittal plane ROM and ankle initial contact angle, the LAS risk increases during impact energy dissipation [41]. Badminton lunges and single-leg landings share similar characteristics, particularly in terms of rapid deceleration and directional change following impact [4,39,41,42]. Given that the lunge places a substantial load on the dominant limb during high-intensity movement and immediate recovery to base position [5,6], players are advised to strengthen the muscles, medial and lateral tissues, and ligaments around the ankle joint to avoid ankle joint injury [39].

In addition to joint moments, this study also examined lower-extremity joint and leg stiffness. Most stiffness-related badminton research has focused on footwear [43,44], leaving joint stiffness and leg stiffness during lunging relatively underexplored. Stiffness can be described as the resistance to deformation of an object in response to an applied force [45]. It arises from the interplay of muscles, tendons, ligaments, cartilage, and bones [46]. Because the badminton lunge is a closed-chain movement involving triple flexion and extension at the hip, knee, and ankle of the dominant limb [47], it can be described within the framework of the stretch-shortening cycle (SSC) [48].

The capacity to develop sufficient lower-limb stiffness is vital for storing elastic energy and generating force during SSC activities [45,49]. The ability to generate higher stiffness in the lower limb benefits movements [34], like maximum-velocity running [50] or changes of direction [51]. The results of this study showed hip stiffness was significantly higher in the forehand lunge, indicating the hip may be adapting to meet greater stability demands in specific directions, aligning with findings that alterations in joint stiffness can help accommodate varying movement speeds or directions [52]. When performing the forehand lunge, maintaining higher hip stiffness may enhance elastic energy storage and concentric force generation at push-off, providing new insight into hip function in directional lunges. The significant changes in hip joint moments and stiffness across different directional lunges may underscore the hip’s important role in controlling movement. Hence, targeted strength training for hip musculature may therefore facilitate more efficient execution of forehand and backhand lunges in amateur badminton.

### Effects of fatigue

Badminton is characterized by actions of short duration and high intensity coupled with short rest periods [2,29,53]. During badminton, participants need to quickly change direction, jump, lunge at the net, and make rapid arm movements from a variety of postural positions [3,54]. Considering these characteristics and combining them with the situation of our laboratory, this study employed a sprint interval running and maximal vertical jump fatigue protocol [30,31] to simulate the fatigue experienced during a match.

Previous studies have reported that greater knee frontal and horizontal plane range of motion (ROM) correlates with better lunge performance in elite and control groups [4,10]. In the present study, we observed a significant reduction in coronal plane knee ROM under fatigue, suggesting that fatigue negatively affects lunge performance. Additionally, we observed a reduction in the peak knee flexion angle during fatigue. This could be due to decreased muscle control (specifically the quadriceps and hamstrings [55,56]) under fatigue, as both concentric and eccentric contractions occur simultaneously [48]. This ultimately limits knee flexion during lunges. A lower knee flexion angle is considered a risk factor for non-contact anterior cruciate ligament (ACL) injuries [57]. As knee flexion decreases and approaches extension, the “screw-home mechanism” activates, tightening the ACL and joint capsule, which increases knee joint loading [58–60]. Overloading the knee joint can lead to injuries affecting the ACL, collateral ligaments, and the meniscus [40,61]. These findings indicate that fatigue not only impairs lunge performance but also potentially increases the risk of knee injuries. Additionally, appropriate increases in ankle sagittal plane ROM and initial contact angles may reduce lower-limb injury risk, particularly for ACL injuries [39]. Therefore, amateur badminton players are advised to strengthen the muscles surrounding the ankle and improve awareness of optimal ankle positioning during lunges to mitigate fatigue-related injury risks.

Earlier, we highlighted the hip joint’s vital role in executing lunges across different directions. However, in this study, we found that during fatigue, the peak hip internal rotation angle and range of motion in the transverse plane decreased. This suggests that fatigue may be a significant factor contributing to decreased movement performance. Specifically, the reduced hip joint activity under fatigue could further affect the performance of lunges in different directions, ultimately impacting badminton performance.

Moreover, higher levels of lower limb stiffness have been reported with increasing force and speed demands during hopping [62], sprinting [63], and endurance running [63]. Consequently, increasing lower limb stiffness could enhance performance in activities involving high ground reaction forces upon impact [45]. In this study, we found that leg stiffness increased significantly during the recovery phase of the lunge under fatigue. This rise in stiffness may serve as a compensatory mechanism, as reduced physical function from fatigue prompts the body to stiffen the lower extremities to meet the force and speed demands. While high levels of stiffness may be beneficial for athletic performance, they are also associated with a greater risk of lower extremity injury [45,64]. Therefore, maintaining proper stiffness becomes crucial for optimizing performance while mitigating injury risks in fatigued states.

### Effects of gender and Interaction between gender and lunge direction

Males generally outperform females in muscular power, strength, and endurance due to the effects of testosterone [14,65,66]. However, there are still gaps in our understanding of how these biological differences impact athletic performance [67].

Previous studies indicate that players can experience impact loads up to 2.5 times their body weight when lunging, necessitating sufficient muscle activity to stabilize the lower limbs [2]. In this study, we found that participants’ impact loads during the lunge were up to 1.7 times their body weight, likely due to suboptimal technique in amateur players. Notably, females exhibited higher secondary impact peaks in ground reaction force and knee internal rotation moments than males. Elevated vertical and horizontal impact forces, especially in early contact phases, produce large joint moments in the lower limb. When excessive, they can contribute to injuries, such as patellar tendinopathy and ACL pathology [17,35,61]. Thus, our findings suggest that females bear a heavier lower limb load during lunging and may face higher knee injury risks. These gender differences align with studies on cutting [68] and landing movements [69], further supporting that female athletes are more prone to ACL injury under higher knee loads. In contrast, male participants in this study exhibited significantly greater peak ankle varus angles and greater range of motion in the coronal plane compared to females. Increased ankle inversion angles are recognized as critical biomechanical contributors to lateral ankle sprain (LAS) risk [37,38,40]. These results indicate that while females may be more susceptible to knee injuries, male players may face a greater risk of LAS during lunging actions due to higher inversion and coronal plane motion.

We also observed that males had higher peak hip adduction moments, whereas females displayed greater hip external rotation moments. Moreover, the interaction between gender and lunge direction revealed that females demonstrated a higher hip transverse plane range of motion in backhand lunges than forehand lunges, whereas males exhibited greater knee coronal plane range of motion in backhand lunges. These differences likely reflect the distinct anatomical and muscular distributions in males and females when performing lunges in various directions.

Specifically, the pelvis is the part of the adult skeleton with the greatest gender-related differences. Female pelvises are wider, with a smaller sacral angle, resulting in a larger Q angle (the angle between the knee and hip joint). A larger Q angle is associated with a higher risk of non-contact knee injuries, such as patellofemoral pain, patellar dislocation, instability, and anterior cruciate ligament (ACL) injuries [70,71]. This anatomical structure may contribute to the greater hip range of motion observed in females during the lunge. In contrast, males have longer pelvises, more curved sacra, and narrower subpubic angles [72,73], which may influence their movement patterns and produce a greater knee ROM during lunges.

Overall, our findings indicate that male and female amateur players adopt distinct biomechanical strategies during forward lunges in different directions. Females appear to rely more on hip flexibility, likely compensating for relatively lower-limb strength, whereas male athletes tend to utilize greater knee musculature. Prior research indicates differences in hip function may stem from weaker hip abductors and external rotators in females [74]. Importantly, strength training has been shown to not only enhance movement quality but also reduce injury risk [75]. Therefore, training programs should be tailored to address the specific biomechanical needs of each gender. In particular, females may benefit from strengthening the muscles surrounding the hip and knee to improve lunge performance and mitigate injury risks. Conversely, males may benefit more from targeted strengthening of the ankle musculature and stabilizing structures to reduce the likelihood of lower-limb injuries.

This study has several limitations. First, all participants were amateur badminton players enrolled at a sports university, suggesting that the findings may be more applicable to amateur populations and may not fully generalize to elite or professional athletes. Second, the fatigue protocol—based on sprint interval running and maximal vertical jumps—may not accurately replicate the specific fatigue experienced during competitive badminton matches. Third, the absence of electromyography (EMG) data limits the ability to assess muscle activation patterns, which are crucial for understanding underlying biomechanical mechanisms. Future research should consider recruiting professional badminton players and designing sport-specific fatigue protocols to enhance the ecological validity of the findings. Additionally, incorporating EMG measurements could offer deeper insights into muscle coordination during lunges, which could provide more comprehensive guidance for injury prevention and training optimization.

## Conclusion

Different lunge directions significantly influenced the joint moment, stiffness, and range of motion of the hip, knee, and ankle, indicating distinct movement control demands in amateur badminton players. Backhand lunges were associated with increased ankle inversion angles and moments, which may elevate the risk of lateral ankle sprains. Under fatigue, reductions in knee joint range of motion and peak flexion angle were observed, potentially increasing anterior cruciate ligament loading. Additionally, increased leg stiffness during recovery may further exacerbate lower limb loading. Gender differences revealed that female players experienced higher knee joint loads, making them more susceptible to knee injuries, while male players exhibited greater ankle inversion, and may face a higher risk of ankle sprains. The interaction between gender and lunge direction highlighted distinct movement strategies, with female athletes potentially benefiting more from hip and knee strengthening to improve performance and reduce injury risk. Based on these findings, training programs should be tailored to address specific biomechanical demands.

## Supporting information

S1 File Provides supporting information for Tables 1–4, respectively.
(XLSX)
